# A Plain Treatise on Dental Science and Practice

**Published:** 1851-10

**Authors:** 


*A Plain Treatise on Dental Science and Practice.* By S. M. Shepherd.

This is a small work intended for private distribution, and for popular instruction, and of course out of the reach of a review. But we are pleased to acknowledge its receipt, and the compliment intended in its dedication to our confrère. It contains a great many good things, and will no doubt be highly beneficial in imparting such knowledge, the possession of which, will prevent many injurious consequences to health and happiness.

Ignorance is a terrible opponent to science and professional skill, and the ardent mother of all empiricism. Any means calculated to reduce this evil is highly acceptable, and must be encouraged. We trust the Dr. will make himself more generally known by contributing to our public mediums such facts as we see he is highly competent to treat upon, and can guarantee him attentive readers. B.

